# Cardiopulmonary Exercise Test in Patients with Hypertrophic Cardiomyopathy: A Systematic Review and Meta-Analysis

**DOI:** 10.3390/jcm10112312

**Published:** 2021-05-25

**Authors:** Adrián Bayonas-Ruiz, Francisca M. Muñoz-Franco, Vicente Ferrer, Carlos Pérez-Caballero, María Sabater-Molina, María Teresa Tomé-Esteban, Bárbara Bonacasa

**Affiliations:** 1 Human Physiology Area, Faculty of Sport Sciences, University of Murcia, Santiago de la Ribera-San Javier, 30720 Murcia, Spain; adrian.bayonas@um.es; 2 Cardiology Department, Virgen de la Arrixaca University Hospital, 30120 Murcia, Spain; fca.mariamf@gmail.com; 3 Physiotherapy Department, Faculty of Medicine, Campus of Espinardo, University of Murcia, 30100 Murcia, Spain; vferrer@um.es; 4 Sports Activities Service, Campus of Espinardo, University of Murcia, 30100 Murcia, Spain; carlospc@um.es; 5 Inherited Cardiopathies Unit, Virgen de la Arrixaca University Hospital, El Palmar, 30120 Murcia, Spain; mariasm@um.es; 6 Cardiovascular Clinical Academic Group, Inherited Cardiovascular Disease Unit, St George’s Hospital NHS Foundation Trust, St George’s University of London, London SW17 0QT, UK; mtome@sgul.ac.uk

**Keywords:** hypertrophic cardiomyopathy, physical activity, exercise test, pathophysiology, prognosis

## Abstract

Background: Patients with chronic diseases frequently adapt their lifestyles to their functional limitations. Functional capacity in Hypertrophic Cardiomyopathy (HCM) can be assessed by stress testing. We aim to review and analyze the available data from the literature on the value of Cardiopulmonary Exercise Test (CPET) in HCM. Objective measurements from CPET are used for evaluation of patient response to traditional and new developing therapeutic measurements. Methods: A systematic review of the literature was conducted in PubMed, Web of Science and Cochrane in Mar-20. The original search yielded 2628 results. One hundred and two full texts were read after the first screening, of which, 69 were included for qualitative synthesis. Relevant variables to be included in the review were set and 17 were selected, including comorbidities, body mass index (BMI), cardiac-related symptoms, echocardiographic variables, medications and outcomes. Results: Study sample consisted of 69 research articles, including 11,672 patients (48 ± 14 years old, 65.9%/34.1% men/women). Treadmill was the most common instrument employed (*n* = 37 studies), followed by upright cycle-ergometer (*n* = 16 studies). Mean maximal oxygen consumption (VO2max) was 22.3 ± 3.8 mL·kg^−1^·min^−1^. The highest average values were observed in supine and upright cycle-ergometer (25.3 ± 6.5 and 24.8 ± 9.1 mL·kg^−1^·min^−1^; respectively). Oxygen consumption in the anaerobic threshold (ATVO2) was reported in 18 publications. Left ventricular outflow tract gradient (LVOT) > 30 mmHg was present at baseline in 31.4% of cases. It increased to 49% during exercise. Proportion of abnormal blood pressure response (ABPRE) was higher in severe (>20 mm) vs. mild hypertrophy groups (17.9% vs. 13.6%, *p* < 0.001). Mean VO2max was not significantly different between severe vs. milder hypertrophy, or for obstructive vs. non-obstructive groups. Occurrence of arrhythmias during functional assessment was higher among younger adults (5.42% vs. 1.69% in older adults, *p* < 0.001). Twenty-three publications (9145 patients) evaluated the prognostic value of exercise capacity. There were 8.5% total deaths, 6.7% cardiovascular deaths, 3.0% sudden cardiac deaths (SCD), 1.2% heart failure death, 0.6% resuscitated cardiac arrests, 1.1% transplants, 2.6% implantable cardioverter defibrillator (ICD) therapies and 1.2 strokes (mean follow-up: 3.81 ± 2.77 years). VO2max, ATVO2, METs, % of age-gender predicted VO2max, % of age-gender predicted METs, ABPRE and ventricular arrhythmias were significantly associated with major outcomes individually. Mean VO2max was reduced in patients who reached the combined cardiovascular death outcome compared to those who survived (−6.20 mL·kg^−1^·min^−1^; CI 95%: −7.95, −4.46; *p* < 0.01). Conclusions: CPET is a valuable tool and can safely perform for assessment of physical functional capacity in patients with HCM. VO2max is the most common performance measurement evaluated in functional studies, showing higher values in those based on cycle-ergometer compared to treadmill. Subgroup analysis shows that exercise intolerance seems to be more related to age, medication and comorbidities than HCM phenotype itself. Lower VO2max is consistently seen in HCM patients at major cardiovascular risk.

## 1. Introduction

Hypertrophic cardiomyopathy is the most frequent and best characterized inheritable cardiac disease (1:500 general population). It is defined by the presence of left ventricular hypertrophy (LVH), measured as thickness of ≥15 mm in adults, in the absence of other loading conditions [1,2]. HCM causes significant morbidity and mortality, and its natural history includes the development of atrial and ventricular arrhythmias such as atrial fibrillation or flutter, and heart failure, stroke, and sudden cardiac death. SCD is the most frequent severe event with a 1% annual incidence [2,3]. In some developed countries, HCM is the most important cause of SCD among young people and athletes [3].

A series of clinical parameters associated with an increased risk of SCD during the follow-up of these patients have been identified. These include the magnitude of the LVH, severity of the left ventricular outflow tract gradient, left atrial diameter, familiar history of syncope or SCD, and the non-sustained ventricular tachycardia (NSVT) in the ambulatory ECG monitoring [4,5,6,7] but also ABPRE and the presence of ventricular arrhythmias. While every parameter has low predictive value when considered alone, risk scores derived from population size sample of patients have been developed [4,5]. HCM patients’ response to exercise has been largely studied. Values of VO2max and ATVO2 have been found reduced in some series. When functional limitation is present, this can be explained related to a reduction in the stroke volume, the chronotropic reserve, an imbalanced ventilation perfusion, and an inadequate peripheral oxygen utilization. 

Patients with chronic diseases frequently adapt their lifestyles to the functional limitations associated with their pathology. Exercise testing in HCM patients is a useful tool to reveal subclinical symptoms and provides valuable information for exercise prescription [8]. HCM patients’ response to exercise has been widely investigated and even proposed as one of the differential diagnostic variables between cases of physiological hypertrophy in athletes and HCM [9,10].

While a comprehensive assessment of functional capacity with ergometry, VO2max analysis with echocardiography allows a better understanding of the pathophysiology in HCM, the diversity of protocols prevents from comparisons between studies. Dyspnea, chest pain, and syncope on exertion are a consequence of the complex involvement of cardiac and extracardiac factors. LVOT together or not with mitral regurgitation, diastolic dysfunction, myocardial ischemia, rhythm disorders, or autonomic imbalance, are the main cardiac factors involved in symptoms development. 

The main aim of the study was to present a systematic review and a meta-analysis of the of available data on cardiopulmonary exercise functional assessment of patients with HCM. The analysis included the evaluation of methodological protocols and the impact of relevant clinical characteristics such as age group, obstruction severity, degree of hyper-trophy and the prognostic implications of the results of the functional tests.

## 2. Experimental Section

### 2.1. Eligibility Criteria

Publications indexed in the Journal Citation Report (JCR) with cross-sectional, case–control, cohorts, randomized controlled trial, clinical trial or case study designs were included. Manuscripts had to be written in English, without restrictions regarding publication date. Systematic reviews, meta-analysis and narrative reviews were not included. According to PICOS strategy, investigations had to assess maximal functional capacity in hypertrophic cardiomyopathy patients in absence of other cardiac diseases. Exercise cessation criteria had to be at least one among event development, limiting symptoms or fatigue impeding continuation. Interventions based on pharmacological treatment, exercise training, surgery, alcohol septal ablation, lifestyle modifications or the combination of them were included. Comparation groups considered for inclusion were healthy controls, athletes, or patients with or without LVOT. Finally, studies without comparation group describing some aspect related to the pathology and accomplishing the aforementioned criteria were considered as well.

### 2.2. Search Strategy

A systematic review of the literature was conducted on PubMed, Web of Science and Cochrane in March 2020. Topics for the search were set throughout a brainstorming session, including keywords according three main areas: HCM, physical exercise and functional capacity: hypertrophic cardiomyopathy, cardiac rehabilitation, physical exercise, exercise tolerance, exercise capacity, anaerobic threshold, maximal oxygen consumption, athlete, assessment, evaluation e intervention. Final search was (((((physical activity[Title]) OR physical exercise[Title]) AND intervention[Title]) OR trial[Title]) OR random*[Title]) AND hypertrophic cardiomyopathy[Title]; and (hypertrophic cardiomyopathy[Title]) AND (physical activity OR physical exercise OR aerobic capacity OR athlete OR anaerobic threshold OR maximal oxygen consumption) AND (assessment OR evaluation OR test *).

### 2.3. Data Extraction and Synthesis

Relevant variables to be included in the review were set and 17 were selected: sample size, age, gender, inclusion of control group, assessment of extracardiac variables, management of pharmacological therapy, material used for the functional assessment, initial intensity and its augments, time lapse for each intensity stage, VO2max, ATVO2, presence of LVOT > 30 mmHg, mitral regurgitation, pulmonary capillary wedge pressure (PCWP), ABPRE and occurrence of cardiac events.

When data were reported in different units, they were synthesized into equal magnitudes according to formulas mentioned for each case. When values were not explicitly indicated, they were calculated from other available data when possible. When BMI was not reported but so were height and weight, it was determined as follows: BMI = weight/height^2^ [11]. When previous fasting was ≥12 h it was considered together with those reported as ‘overnight’. When speed (V) was reported in miles per hour (mph) it was transformed into kilometers per hour (km·h^−1^) as follows: Vkm·h^−1^ × 1.609. When maximal aerobic capacity was expressed in metabolic equivalents (METs), it was recalculated to ml·kg^−1^·min^−1^ of oxygen consumption as previously indicated [12], i.e., a maximal work rate reached at 7 METs on a cycle-ergometer multiplied by 3.5 to be recalculated into 24.5 mL·kg^−1^·min^−1^.

### 2.4. Statistical Analyses

Global mean values and standard errors were calculated as the summatory of the product of each class mark multiplied by its frequency and divided by the sample size. The prevalence of extracardiac variables and inclusion of the assessment of cardiac variables in the studies was evaluated as the summatory of cases divided by the sample size. Random effects meta-analyses and subgroup analyses were performed using Review Manager (RevMan), V.5.3. Copenhagen: The Nordic Cochrane Centre, The Cochrane Collaboration, 2014). Differences in the prevalence of ABPRE and arrhythmia before and during exercise were analyzed using a Chi-Square test on SPSS v.23. Alpha was set on 0.05.

## 3. Results

### 3.1. Study Sample

**Figure 1:**
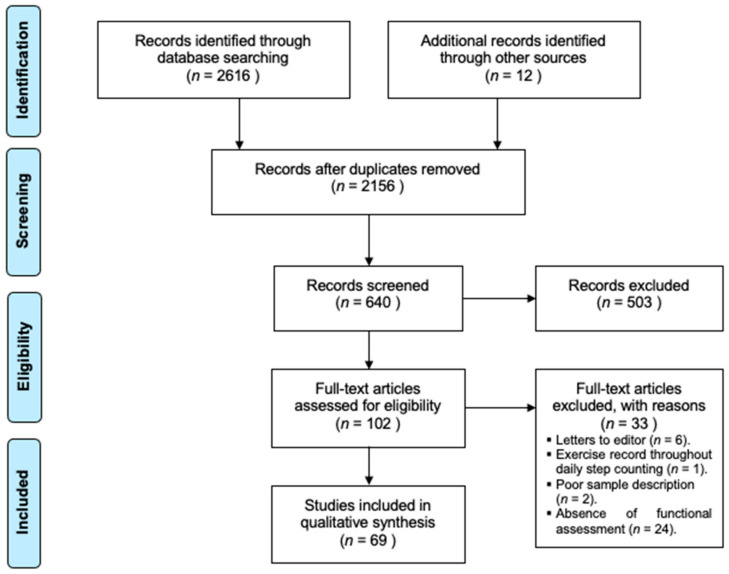
Flow diagram of the search process. Adapted from Moher and collaborators [13].

Original search yielded 2616 results and 12 more were added based on the exploration of the references of systematic reviews and meta-analyses related to hypertrophic cardiomyopathy. When full texts were not available online, authors were contacted after an initial screening for title and abstract. A flow diagram of the search process is available in Figure 1. One hundred and two full texts were read after the first screening, of which, 69 were included for qualitative synthesis. The protocol was designed ad hoc and not published. All data concerning methodological designs are showed in Table 1. They included 11,672 patients which were 48 ± 14 years old and had an average body mass index of 26.9 ± 6.5 kg/m^2^ as evaluated with data from 21 studies and 6558 patients. In total, 65.9% of them were men and 34.1% women. In 18 of the 69 articles, at least one extracardiac variable was included, among which the presence of diabetes and pathologies involving the lipidic metabolism were the most common with 14 and 7 times reported, respectively. The presence of diabetes was calculated with data from 3665 patients with a prevalence of 7.6%; and from 1927 patients for dyslipidemias with a prevalence of 45.1%. Other comorbidities such as hypertension and smoking habit were present in 32.8% and 30.6% of patients, respectively.

**Table 1:** Methodological characteristics of publications.

Author, Year	*n*	Age (Years)	%W	CPETMaterial	CPET Intensity			Drug Therapy	Extracardiac Variables	Cardiac Variables Measured				
					Initial	Added	Lapse			LVOT	MR	PCWP	ABPRE	Events
Abozguia, 2010 [14]	56	52 ± 11	29	Treadmill	4.7 M	2–3 M	3′	Not controlled	-	Out	-	-	-	-
Aljaroudi, 2012 [15]	495	50 ± 15	32	Treadmill	4.7 M	2–3 M	3′	Disc. (12 h)	DM, HL	BL	BL	-	-	Out
Arena, 2008 [16]	83	38 ± 10	39	Upright CE	15 W	15 W	1′	Continued	-	Out	Out	BL, Ex	-	-
Austin, 2010 [17]	50	44 ± 13	38	Treadmill	-	-	-	Continued	-	BL, Ex	BL	-	Ex	-
Axelsson, 2016 [18]	130	52 ± 13	35	Upright CE	25 W	25 W	2′	Continued	-	BL	-	-	-	BL
Azarbal, 2014 [19]	265	52 ± 15	39	Treadmill	2 mph	-	-	Continued	DM, HL	BL	BL	-	Ex	-
Binder, 2007 [20]	217	56 ± 16	41	-	-	-	-	Not registered	Creatinine	BL	BL	-	-	-
Bonow, 1985 [21]	70	47	47	Treadmill	2 mph	2.5%	2′	Administered	-	BL	-	-	-	Ex
Briguori, 1999 [22]	52	41	29	Upright CE	60 rpm	1 W	3″	Disc. (5H-L)	-	BL	BL	-	Ex	Ex
Briguori, 2001 [23]	44	40 ± 15	27	(?) CE	60 rpm	1 W	3″	Disc. (5H-L)	Blood analysis	BL	BL	-	-	Ex
Chan, 1990 [24]	13	42 ± 14	38	Upright CE	28 W	22 W	3′	Administered	-	BL	-	BL	-	Out
Chikamori, 1992 [25]	81	41	46	Treadmill	4.7 M	2–3 M	3′	Disc. (?)	-	BL	Out	-	Out	Out
Choi, 2008 [26]	32	55 ± 11	19	Supine CE	25 W	25 W	3′	Disc. (?)	Creatinine, HL	BL	Out	-	-	-
Ciampi, 2007 [27]	22	36 ± 13	23	Treadmill	2.7 M	2–3 M	3′	Disc. (5H-L)	-	BL	-	-	Ex	Ex
Coats, 2014 [28]	1898	47 ± 15	33	Upright CE	0 W	10–30W	1′	Continued	-	BL	BL	-	Ex	Ex
D’Andrea, 2017 [29]	45	38 ± 15	22	Supine CE	-	25 W	2′	No drugs used	-	BL	-	BL, Ex	-	-
de la Morena, 2003 [30]	98	44 ± 15	28	Treadmill	4.7 M	2–3 M	3′	Disc. (48 h)	-	BL	BL	-	Ex	Ex
de la Morena, 2013 [31]	87	54 ± 13	33	Treadmill	4.7 M	2–3 M	3′	Not controlled	-	BL, Ex	BL, Ex	-	Ex	Ex
Desai, 2014 [32]	426	44 ± 14	22	Treadmill	4.7 M	2–3 M	3′	Continued	DM	BL, Ex	BL, Ex	-	Ex	Ex
Dimitrow, 1996 [33]	10	37 ± 7	-	Treadmill	2.7 M	2–3 M	3′	Administered	DM, HL	BL	-	-	-	-
Dumont, 2007 [34]	64	51 ± 14	33	Treadmill	4.7 M	2–3 M	3′	Disc. (72 h)	-	BL	-	-	Ex	BL
Efthimiadis, 2011 [35]	68	45 ± 15	34	Treadmill	4.7 M	2–3 M	3′	Not controlled	-	BL	-	-	Ex	BL
Ferguson, 2016 [36]	22	14 ± 3	44	Upright CE	25 W	25 W	3′	Continued	-	BL, Ex	-	-	-	-
Finocchiaro, 2015 [37]	156	51 ± 14	38	Treadmill	-	-	-	Continued	-	BL	BL	-	-	-
Frenneaux, 1989 [38]	23	30	39	Treadmill	2.7 M	2–3 M	3′	Continued	-	BL	Out	BL, Ex	-	BL
Ghiselli, 2019 [39]	292	46 ± 16	28	Semisup CE	-	25 W	2′	Continued	DM	BL, Ex	BL, Ex	-	Ex	BL, Ex
Ha, 2005 [40]	29	57 ± 10	16	Supine CE	25 W	25 W	3′	Disc. (?)	DM	-	-	-	-	-
Hernández, 2015 [41]	40	55 ± 12	36	Treadmill	2.7 M	2–3 M	3′	Not registered	DM	BL	-	-	-	-
Jones, 1998 [42]	50	35 ± 14	30	Upright CE	0 W	5–15 W	1′	Disc. (48 h)	-	BL	-	-	Out	Ex
Kim, 2004 [43]	21	49 ± 15	43	Treadmill	4.7 M	2–3 M	3′	Administered	-	BL	-	-	-	-
Kitaoka, 2009 [44]	31	52 ± 17	35	Upright CE	0 W	15 W	1′	Continued	-	BL	-	-	Ex	-
Kjaergaard, 2005 [45]	99	49 ± 15	34	Treadmill	2 mph	2 M	2′	Continued	-	BL	BL	Out	-	-
Konecny, 2015 [46]	198	53 ± 16	38	Treadmill	2.5 M	2,5 M	2′	Continued	DM, ∑skinfold	BL	BL	-	-	-
Lafitte, 2013 [47]	107	52 ± 15	33	Semisup CE	-	-	-	Continued	-	BL, Ex	BL	-	-	Ex
Larsen, 2018 [48]	510	51 ± 15	36	Treadmill	-	2 M	2′	Disc. (?)	-	BL	BL	-	-	BL
Le, 2009 [49]	63	48 ± 15	28	Treadmill	-	-	-	Continued	DM, HL	BL, Ex	BL, Ex	-	Ex	Ex
Lele, 1995 [50]	23	30	39	SupC + Tread	25 W	12.5 W	3′	Discont. (120 h)	-	BL	BL	BL, Ex	Ex	BL, Ex
Lösse, 1983 [51]	122	42 ± 3	-	Supine CE	-	-	-	Administered	-	BL, Ex	-	BL, Ex	Ex	-
Luo, 2015 [52]	273	50 ± 15	30	Treadmill	2.7 M	2–3 M	3′	Not registered	-	BL, Ex	-	-	Ex	Ex
Ma, 2014 [53]	27	54 ± 12	42	Treadmill	2.7 M	2–3 M	3′	Not registered	DM	BL, Out	BL, Out	-	-	-
Magri, 2014 [54]	180	47 ± 16	25	Upright CE	0 W	-	-	Continued	-	BL	-	-	-	-
Magri, 2016 [55]	683	49 ± 16	31	Supine CE	-	-	-	Continued	DM	BL	-	-	Ex	Ex
Magri, 2018 [56]	767	48 ± 16	32	(?) CE	0 W	5–15 W	-	Not controlled	-	BL	-	-	Ex	BL, Ex
Malek, 2009 [57]	13	-	-	Treadmill	-	-	-	Not registered	-	-	-	-	-	-
Matsumoto, 2005 [58]	27	35	33	Treadmill	-	0.5%	1′	Continued	-	BL, Out	BL	-	-	-
Matsumura, 2002 [59]	85	38 ± 14	34	Upright CE	0 W	5–15 W	1′	Disc. (5H-L)	-	BL	Out	-	-	-
Menon, 2008 [60]	88	14	25	Treadmill	2.7 M	2–3 M	3′	Not registered	-	BL	BL	-	-	-
Miki, 1990 [61]	22	47 ± 15	18	Treadmill	2.7 M	2–3 M	3′	Disc. (120 h)	-	-	-	BL, Ex	-	-
Mizukoshi, 2013 [62]	33	59 ± 16	27	Semisup CE	20 W	1–2 W	6″	Continued	-	Out	-	-	-	Ex
Moneghetti, 2017 [63]	131	52 ± 13	37	Treadmill	-	-	-	Not controlled	-	BL	BL	-	Ex	BL
Nihoyannopoulos, 1992 [64]	40	41	45	Treadmill	2.7 M	2–3 M	3′	Continued	-	BL	BL	-	-	-
Nistri, 2010 [65]	74	45 ± 16	28	Upright CE	-	25 W	2′	Disc. (5H-L)	-	BL, Ex	BL	-	-	Ex
Payá, 2008 [66]	120	47 ± 16	30	Treadmill	4.7 M	2–3 M	3′	Not registered	-	BL	-	-	Ex	BL
Peteiro, 2012 [67]	239	52 ± 15	39	Treadmill	2.7 M	2–3 M	3′	Continued	DM, HL	BL, Ex	BL, Ex	-	Ex	BL, Ex
Pozios, 2018 [68]	95	49 ± 16	31	Treadmill	2.7 M	2–3 M	3′	Continued	DM, HL	BL, Ex	-	-	-	Ex
Re, 2017 [69]	197	45 ± 15	35	Upright CE	-	10W	1′	Disc. (72 h)	-	BL, Ex	BL	-	Ex	Out
Romero, 2008 [70]	98	46 ± 15	29	Treadmill	2.7 M	2–3 M	3′	Not registered	-	BL	-	-	Ex	BL
Rosing, 1979 [71]	27	44 ± 3	41	Treadmill	2 mph	2.5%	2′	Administered	-	BL	-	BL	-	-
Sachdev, 2005 [72]	43	36 ± 10	40	Upright CE	-	15 W	-	Not registered	-	Out	BL	BL, Ex	-	-
Saura, 2009 [73]	75	46 ± 14	25	Treadmill	4.7 M	2–3 M	3′	Disc. (48 h)	DM	BL	-	-	Ex	BL
Shah, 2019 [74]	9	29 ± 8	0	Upright CE	0 W	20 W	1′	Continued	Blood analysis	Out	-	-	-	Ex
Shizukuda, 2005 [75]	49	36 ± 10	41	Upright CE	-	5 W	-	Not registered	-	Out	-	BL, Ex	-	-
Smith, 2018 [76]	589	51 ± 14	39	Treadmill	-	-	-	Continued	-	BL	-	-	-	-
Smith, 2018 [77]	1177	53 ± 14	43	Treadmill	-	2 M	2′	Continued	DM, HL, Hb	BL	-	-	Ex	-
Thaman, 2006 [78]	171	46 ± 18	63	Upright CE	-	10–25W	1′	Continued	-	BL	-	-	-	BL
Wu, 2019 [79]	67	53 ± 11	12	Semisup CE	25 W	25 W	2′	Disc. (24 h)	-	Out	-	-	-	-
Wu, 2019 [80]	88	52 ± 12	14	Semisup CE	25 W	25 W	2′	Disc. (24 h)	-	BL	-	-	-	-
Wu, 2019 [81]	76	48 ± 12	20	Semisup CE	25 W	25 W	2′	Disc. (24 h)	-	BL, Ex	-	Ex	Ex	-
Yetman, 2001 [82]	17	12 ± 3	30	Treadmill	4.7 M	2–3 M	3′	Not controlled	-	BL, Ex	BL	-	-	-

%W: percentage of women in the sample; CPET: cardiopulmonary exercise test; BL: baseline measure; EX: exercise measure; Out: exclusion criteria; CE: cycle-ergometer; Sup CE: supine cycle-ergometer; Semisup CE: semi-supine CE; Tread: treadmill; (?) CE: cycle-ergometer with unknown position of the patient; 5H-L: 5 half-lives; Disc.: drug therapy withdrawn a certain time before the test; Discont. (?): drug therapy withdrawn before the test but no further information about time available; ‘M’: METs; DM: diabetes mellitus; HL: hyperlipidemia; ∑skinfolds: summatory of three skinfolds; Hb: hemoglobin content. ‘Not controlled’: drug use reported without information about its management. When added intensity is reported with ‘%’, it means that slope of the treadmill was augmented in that certain percentage.

Management of the pharmacological therapy showed 12 different options and was evaluated in 60 of the 69 studies. In seven of them, the use of drugs was recorded but no further information about its management was provided. In 26 of the 53 remaining, drugs were not discontinued before the study; in 6, the researchers provided it; and in 1, none of the subjects were on medication. Finally, in the 20 remaining studies, drugs were discontinued with variable time from 5 days to 12 h prior to the exercise test.

Treadmill was the most common ergometer employed (*n* = 37 studies), followed by upright cycle-ergometer (*n* = 16 studies), supine and semi-supine cycle-ergometers (*n* = 6 studies each). Bruce’s protocol [83] and its modified version [84] were the most frequently used, while there was a marked heterogeneity in the protocols employed in the cycle-ergometers. The initial intensity was set between 0 W and 28 W with increments ranging from 5 W to 30 W and lapses lasting 1 to 3 min. There were 2 publications where the intensity was increased in a ramp-like fashion adding 1–2 W each 1–6 s.

### 3.2. Maximal Oxygen Consumption

**Figure 2:**
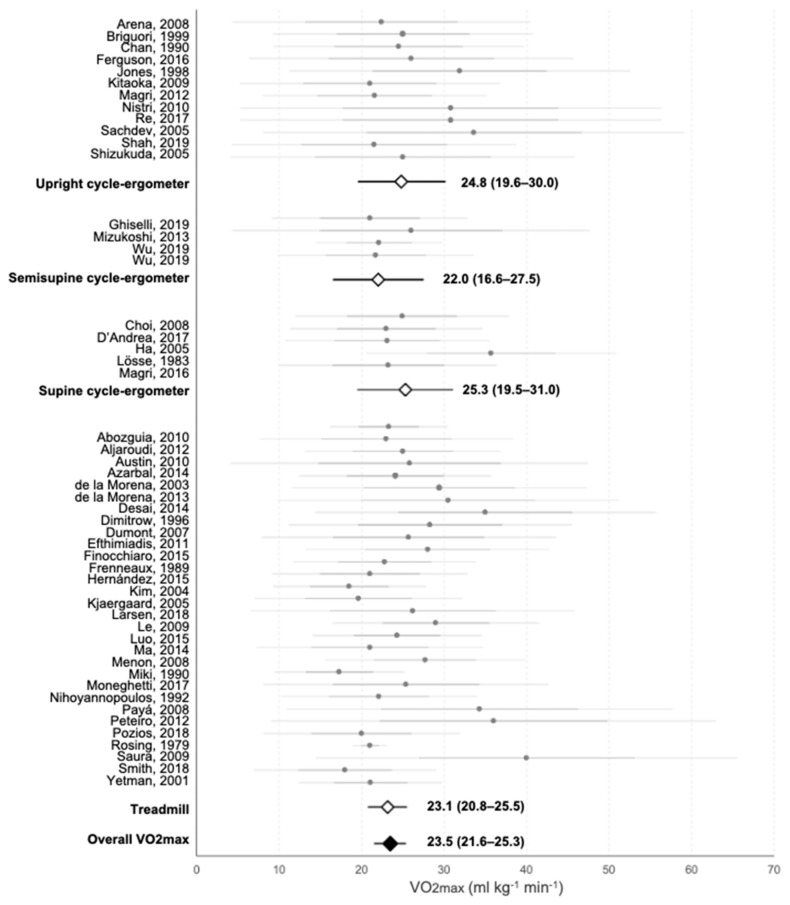
Meta-analysis of the VO2max reported on each study and average values for each ergometer used on the assessment of functional capacity. Grey points represent the average value of each individual study. Darker horizontal lines intersecting them represent the upper and lower value of the mean ± SD, and lighter horizontal lines show 95% CI. White rhombi intersected by a black line represent the mean VO2max and 95% CI of each ergometer as extracted from the meta-analysis. The black rhombus represents the mean value and 95% CI of all studies as extracted from the meta-analysis. Numerical values of each subgroup and the whole group are presented.

**Figure 3:**
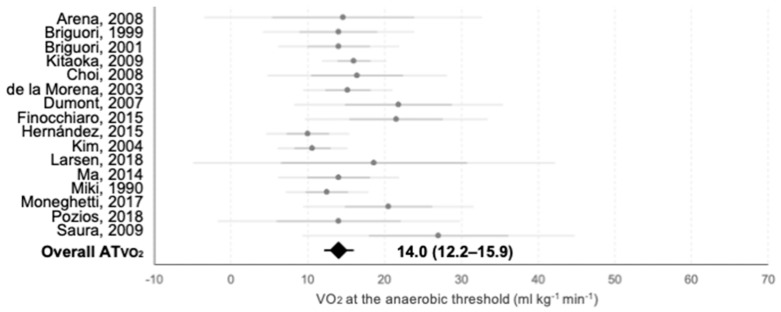
Meta-analysis of the ATVO2 reported on each study. Grey points represent Table 2 of each individual study. Darker horizontal lines intersecting them represent the upper and lower value of the mean ± SD, and lighter horizontal lines show 95% CI. The black rhombus represents the mean value and 95% CI of all studies as extracted from the meta-analysis. Numerical values for the whole group are presented.

Maximal oxygen consumption was reported in 54 of the 69 texts and 51 of them were included in a semi-meta-analysis to calculate the average values achieved in the literature and to elucidate on which ergometer showed higher values. Data from 2 studies were not included because of lack of information about patients’ position on the cycle-ergometer and from 1 study because of lack in standard deviation information. Moreover, ATVO2 was indicated in 18 publications and 15 of them were included in semi-meta-analysis. Data from 3 studies were not included as values of standard deviation were unavailable. Data of each individual study are shown together with the mean values recorded in each ergometer and the average VO2max in Figure 2. Mean VO2max was 22.3 ± 3.8 mL·kg^−1^·min^−1^, while the highest average values were observed in supine and upright cycle-ergometer: (25.3 ± 6.5 and 24.8 ± 9.1 mL·kg^−1^·min^−1^; respectively). Semi-supine cycle-ergometer and treadmill showed lower mean VO2max (22.0 ± 5.3 and 21.9 ± 3.1 mL·kg^−1^·min^−1^; respectively; although differences did not reach significance: *p* = 0.87 for all groups; *p* = 0.57 for comparison between upright cycle-ergometer vs. treadmill). Mean ATVO2 was 14.6 ± 4.5 mL·kg^−1^·min^−1^ and data of each study are shown in Figure 3. According to this, ATVO2 represented a 65.5% of VO2max.

### 3.3. Cardiac Variables

Data regarding cardiac variables were registered as included or non-included together with the moment when measuring was done. Numerical data (not shown) were analyzed separately and will be mentioned hereafter. Average prevalence of resting LVOT > 30 mmHg was 31.4% in 7933 patients and increased to 49% in exercise conditions (data from 1095 patients), meaning 1 out of 3 patients without resting LVOT > 30 mmHg developed obstruction with exercise (287 out of 847).

Mitral regurgitation (MR) was evaluated in 27 publications: 22 at rest and 5 at rest and during exercise. Prevalence of resting MR data was 33.9% (*n* = 5587) with no distinction of the MR grade and augmented up to 62.0% during exercise.

Mean PCWP and pulmonary artery diastolic pressure during exercise was obtained with data from 175 patients (11.9 ± 5.6 and 12.0 ± 5.8 mmHg, respectively), while average values for pulmonary artery systolic pressure and mean pulmonary artery pressure were 28.5 ± 7.7 and 15.8 ± 5.0 mmHg, respectively.

Mean prevalence of ABPRE was 20.3%, but the available data prevented distinctions between ABPRE type (e.g., blood pressure drop, failure in increasing blood pressure). 

Regarding arrhythmias, 1496 (32.7%) patients experienced this event during 24 h Holter monitoring prior to the CPET and 2.67% of them developed it during the test.

### 3.4. Analysis of Subgroups

Information from publications was extracted and sorted regarding age, presence of baseline LVOT, and severity of hypertrophy. 

**Figure 4:**
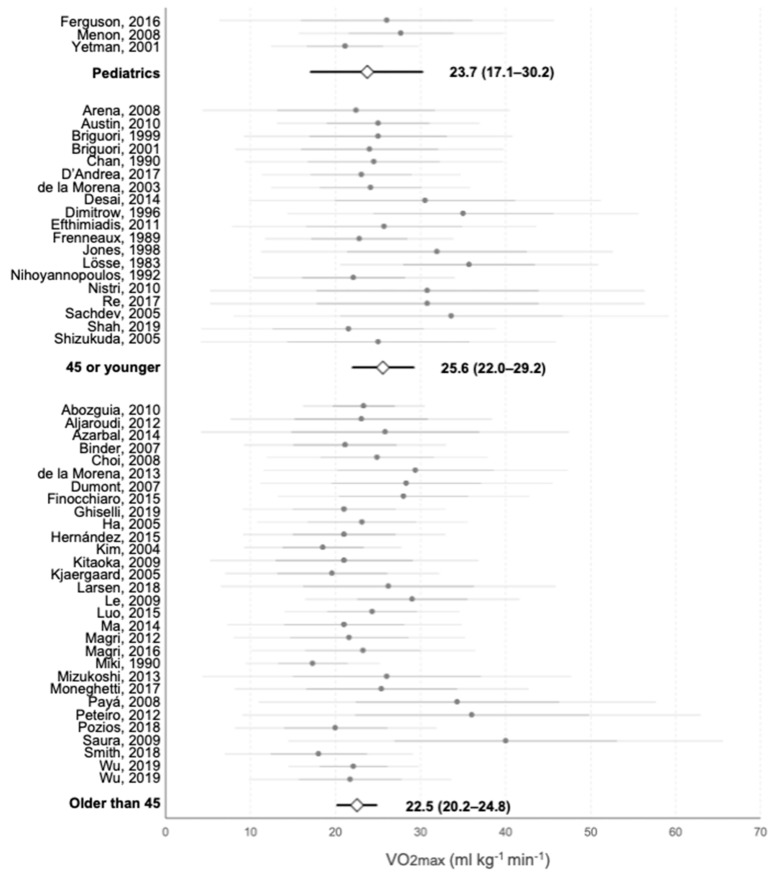
Meta-analysis of the VO2max reported on each study and average values for each age group. Grey points represent the average value of each individual study. Darker horizontal lines intersecting them represent the upper and lower value of the mean ± SD, and lighter horizontal lines show 95% CI. White rhombi intersected by a black line represent the mean VO2max and 95% CI of each age group as extracted from the meta-analysis. Numerical values of each subgroup are presented.

Age cut-offs were arbitrarily set. Figure 4 represents VO2max for three pediatric (*n* = 127), 19 ≤ 45 years old (*n* = 1496) and 30 > 45 years old (*n* = 5088) cohorts extracted information from related publications. Mean VO2max was higher for adults ≤ 45 years old, then pediatric HCM patients and older adults, although differences did not reach significance (25.6 ± 3.6 mL·kg^−1^·min^−1^ vs. 23.7 ± 6.6 mL·kg^−1^·min^−1^ and 22.5 ± 2.5 mL·kg^−1^·min^−1^, respectively, *p* = 0.61 for young adults vs. pediatric, *p* = 0.15 for young vs. older adults). Proportion of ABPRE was higher in young adult and older adult groups compared to pediatric (20.4% vs. 14.2% and 2.75%, respectively, *p* < 0.001 for all groups and for paired comparisons). Prevalence of previous arrhythmias was higher for older adults than for younger adults (26.4% vs. 21.5%, *p* < 0.01), while the occurrence during functional assessment was higher among younger adults (5.42% vs. 1.69% in older adults, *p* < 0.001). There were no data regarding the presence or occurrence of arrhythmias in pediatric patients.

**Figure 5:**
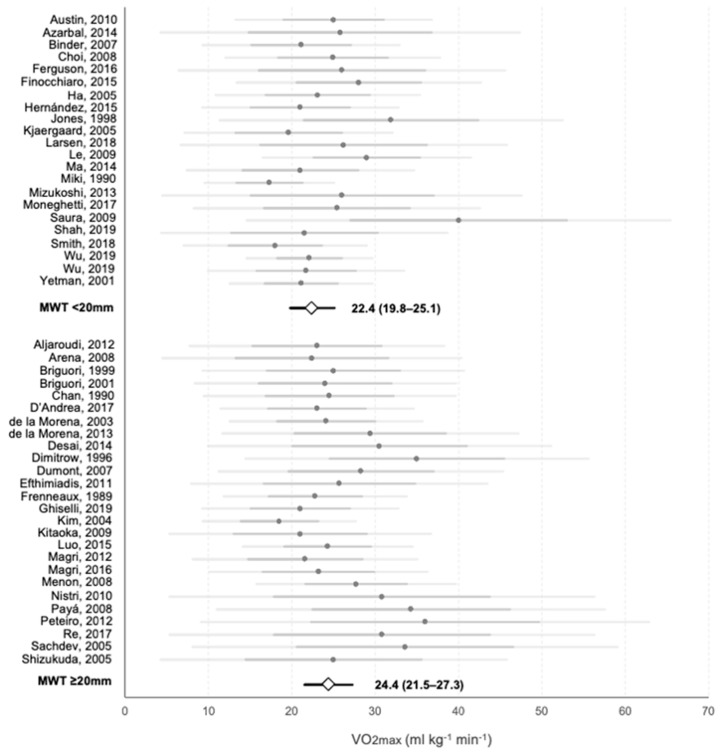
Meta-analysis of the VO2max reported on each study and average values for MWT groups. Grey points represent the average value of each individual study. Darker horizontal lines intersecting them represent the upper and lower value of the mean ± SD, and lighter horizontal lines show 95% CI. White rhombi intersected by a black line represent the mean VO2max and 95% CI of each MWT group as extracted from the meta-analysis. The numerical values of each subgroup are presented.

A 20 mm cut-off, which is close to the mean maximal left ventricular wall thickness for most publications, was arbitrarily set for mean VO2max comparison. Figure 5 represents VO2max results of 22 and 26 publications with available information for under and above 20 mm analysis. A total of 2600 patients were included in the milder and 3798 patients in the group with more severe hypertrophy. The group with more severe hypertrophy had a higher mean VO2max, although differences did not reach significance (24.4 ± 2.9 mL·kg^−1^·min^−1^ vs. 22.4 ± 2.6 mL·kg^−1^·min^−1^, *p* = 0.34). Proportion of ABPRE was higher in severe vs. mild hypertrophy groups (17.9% vs. 13.6%, *p* < 0.001). The prevalence of previous arrhythmias and occurrence during functional assessment was similar in mild versus severe hypertrophy groups (26.5% vs. 24.5%, *p* = 0.44 and 2.79% vs. 2.11%, *p* = 0.26, respectively).

**Figure 6:**
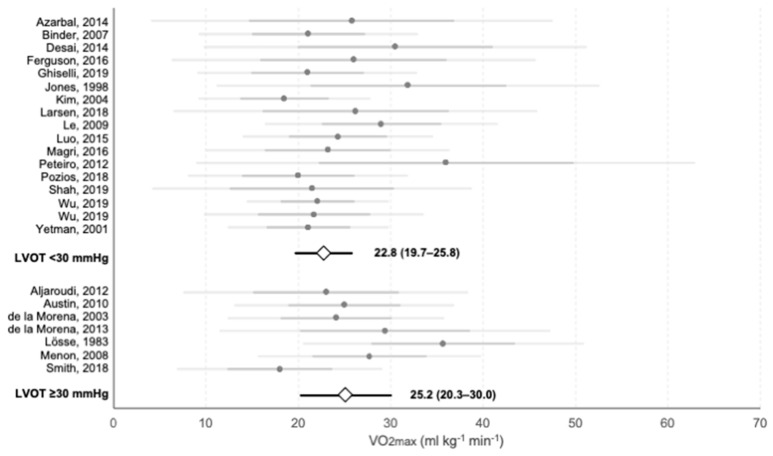
Meta-analysis of the VO2max reported on each study and average values for each LVOT group. Grey points represent the average value of each individual study. Darker horizontal lines intersecting them represent the upper and lower value of the mean ± SD, and lighter horizontal lines show 95% CI. White rhombi intersected by a black line represent the mean VO2max and 95% CI of each LVOT group as extracted from the meta-analysis. The numerical values of each subgroup are presented.

Information on exercise mean VO2max was available for comparison between baseline LVOT ≥ 30 mmHg (*n* = 1529) and LVOT < 30 mmHg (*n* = 3346) and is shown in Figure 6. Mean VO2max was slightly higher for obstructive vs. non-obstructive series, although differences did not reach significance (25.2 ± 4.9 vs. 22.8 ± 3.1 mL·kg^−1^·min^−1^, *p* = 0.41). Proportion of ABPRE was higher in non-obstructive versus obstructive groups (12.8% vs. 7.1%, *p* < 0.001). Prevalence of previous arrhythmias was 30.7% in non-obstructive patients, while no data was available for obstructive groups. Occurrence of arrhythmia during functional assessment was similar in obstructive and non-obstructive groups (5.55% vs. 4.08%, *p* = 0.29).

### 3.5. Prognostic Studies

23 publications evaluating the prognostic value of exercise capacity for prediction of major cardiac complications were identified. A total of 9145 patients were included in this analysis. Table 2 summarizes main characteristics and results of the selected studies. Sixteen studies reported on SCD or equivalent (resuscitated cardiac arrest and ICD therapies), 13 on heart failure death, 7 on stroke-related death, 8 on heart transplantation, 19 on global cardiovascular mortality and 14 on all-cause mortality. There were 8.47% total deaths, 6.72% cardiovascular deaths, 3.06% SCD cases, 1.20% heart failure death, 0.65% resuscitated cardiac arrests, 1.01% transplants, 2.58% ICD therapies and 1.22 strokes. Mean follow-up of the cohorts was 3.81 ± 2.77 years for a total of 43,794 patient years followed. SCD was assessed in 5519 patients and occurred in 232 of them with an incidence of 7.1 cases per 1000 patient^−1^ years^−1^. Appropriate implantable cardioverter defibrillator discharge was present in 110 cases among 4258 patients, with an incidence rate (IR) of 5.4 cases per 1000 patient^−1^ years^−1^. Heart failure-related death was considered in 4594 patients and occurred in 55 of them with an (IR) of 2.4 per 1000 patient^−1^ years^−1^. Resuscitation from sudden death was successfully achieved in 27 out of 4173 patients (IR: 1.3 per 1000 patient^−1^ years^−1^). Stroke occurred in 41 cases among 3361 patients (IR: 2.4 per 1000 patient^−1^ years^−1^). Forty patients had heart transplantation (IR: 2.1 per 1000 patient^−1^ years^−1^). Finally, cardiovascular and all-cause mortality were assessed in 6797 and 5594 patients, with an IR of 13.9 and 16.3 per 1000 patient^−1^ years^−1^, respectively.

**Table 2:** Characteristics of prospective studies.

Author, Year	*n*	Age	%W	F-up	Drug Therapy	Prospective Outcomes (Cases)							
						SCD	ICD	HFm	Res	Str	HT	CVm	All
Caselli, 2014 [85]	25	26	0	4.0	Not controlled	0	-	0	-	-	-	0	0
Coats, 2014 [28]	1898	47	33	5.6	Continued	40	-	31	-	-	22	117	178
Desai, 2014 [32]	426	44	22	8.7	Continued	30	13	7	0	8	-	37	40
Feneon, 2016 [86]	126	47	22	2.4	Continued	-	1	-	3	-	-	7	-
Finocchiaro, 2015 [37]	156	51	38	2.3	Continued	-	-	-	-	-	5	4	-
Ghiselli, 2019 [39]	292	46	28	5.9	Continued	1	-	-	1	13	-	6	-
Gimeno, 2009 [87]	1380	42	38	4.5	Not controlled	-	-	-	-	-	-	158	-
Limongelli, 2019 [88]	51	39	24	1.8	Not controlled	0	-	0	-	-	-	0	0
Magri, 2016 [61]	683	49	31	3.7	Continued	25	18	-	4	-	-	-	-
Magri, 2016 [89]	620	49	31	3.8	Not controlled	25	18	4	3	3	5	36	50
Magri, 2018 [56]	767	48	32	4.2	Continued	23	14	4	5	3	5	30	30
Masri, 2015 [90]	1005	50	36	5.5	Not controlled	-	17	-	2	11	-	-	126
Michaelides, 2009 [91]	81	42	30	5.3	Continued	8	6	0	6	-	-	8	-
Moneghetti, 2017 [63]	131	52	37	4.7	Not controlled	-	-	-	-	-	-	-	6
Nagata, 2003 [92]	65	50	23	6.3	Disc. (24 h)	0	0	-	0	-	0	0	0
Olivotto, 1999 [93]	126	42	29	4.7	Disc. (5H-L)	3	-	6	-	-	-	9	9
Peteiro, 2012 [67]	239	52	39	4.1	Continued	-	2	-	-	1	1	5	-
Peteiro, 2015 [94]	148	51	34	7.1	Continued	0	1	0	1	2	0	0	0
Pozios, 2018 [68]	95	49	31	3.4	Continued	0	18	0	-	-	-	0	0
Reant, 2015 [95]	115	52	34	1.6	Continued	0	-	1	-	-	-	1	-
Sadoul, 1997 [96]	161	27	35	3.7	Disc. (5H-L)	12	2	1	2	-	2	15	17
Smith, 2018 [76]	589	51	39	4.3	Continued	-	-	-	-	-	-	-	-
Sorajja, 2012 [97]	182	53	35	4.0	Continued	2	-	1	-	-	-	10	18

SCD: sudden cardiac death, ICD: implantable cardioverter defibrillator discharge, HFm: heart failure-related mortality, Res: resuscitation, Str: stroke, HT: heart transplantation, CVm: cardiovascular mortality, All: all-cause mortality. Age and follow-up (F-up) are expressed in years. Other abbreviations can be found in Table 1.

**Figure 7:**
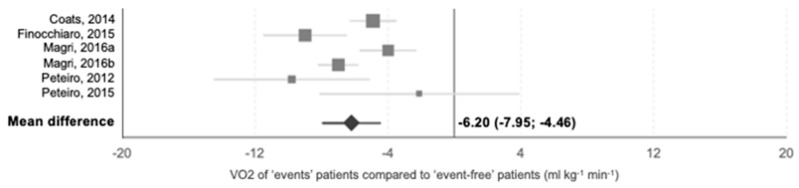
Random-effects meta-analysis of VO2max values of patients with recorded events during follow up compared to event-free patients. Grey squares represent mean difference between groups and its size indicates its relative weight in the meta-analysis. Grey lines intersecting them horizontally represent 95% CI for the mean differences. The black rhombus represents the mean difference of all studies, indicating that ‘events’ patients had lower VO2max than ‘event-free’.

Fourteen publications identified exercise-related predictors of SCD, 13 for heart failure death and 15 for all related mortality. VO2max (*n* = 7 publications), ATVO2 (*n* = 4), METS (*n* = 6), % of age-gender predicted VO2max (*n* = 7), % of age-gender predicted METs (*n* = 1), ABPRE (*n* = 7), and ventricular arrhythmias (*n* = 6) were significantly associated with major outcomes as individual predictors. Individual hazard ratios extracted from each study are presented in Table 3. Furthermore, data regarding functional capacity of groups of patients who suffered from any of the aforementioned events and patients free of events were extracted and meta-analyzed to elucidate differences in VO2max. Only 6 publications had detailed information of 3684 patients concerning this parameter. Mean VO2 max was reduced in patients who reached the combined cardiovascular death outcome compared to those who survived (−6.20 mL·kg^−1^·min^−1^; CI 95%: −7.95, −4.46; *p* < 0.01; Figure 7).

## 4. Discussion

The systematic review of the available data on cardiopulmonary functional assessment of patients with HCM presented here highlights a need for the standardization of the protocols and provides a comprehensive evaluation of the determinants of exercise capacity. The dissection of 69 studies meeting selection criteria, including a large cohort of 11,672 patients, demonstrated the role of objective exercise capacity evaluation for identification of symptomatic limitations. A mean VO2max of 23.5 (21.6, 25.3) ml·kg^−1^·min^−1^ characterizes a disease with a reduction in exercise capacity of at least a 20% from that expected for similar age general population [28,37].

### 4.1. Clinical and Prognostic Value of CPET

Despite not significant differences were seen regarding age groups, presence or absence of obstruction and the degree of hypertrophy in the achieved VO2max, this summary variable (VO2max) demonstrated to have prognostic implications. The group of patients with reported events had −6.20 (−9.95, −4.46) ml·kg^−1^·min^−1^ compared to the group with no events during follow-up (43,794 patient years followed). This association has been consistently demonstrated in several publications [28,37,55,67,89].

The results of the pooled analysis of the largest cohort of patients undergoing cardiopulmonary functional assessment, provides relevant information of the magnitude of different characteristics of this relatively common condition. The proportion of resting obstruction was in the range of previous publications 31% [5,6]. This figure increased to 49% with the inclusion of exercise induced obstruction [6]. The mean prevalence of ABPRE was 20%, with a positive statistical association with the degree of hypertrophy and young adult age. The proportion of ABPRE was unexpectedly significantly lower in the obstructive vs. non-obstructive groups. The pooled analysis presented here provides relevant information on less reported aspects such as the proportion of patients with MR at rest (34%) which parallel to obstruction, increased with exercise to a 62%. Mean pooled PCPW during exercise despite a significant proportion of patients with obstruction and MR seemed to remain relatively low (11.9 ± 5.6 mmHg). The prevalence of arrhythmias during exercise was rarely reported in the range of 2–5% for different group analysis.

Patients with chronic cardiac diseases tend to accommodate their life activities to their physical capacity. CPET has demonstrated to be a safe and objective way to assess clinical limitations to exercise in HCM patients [98]. The risk of developing severe arrhythmic complications such as sustained ventricular tachycardia or fibrillation during exercise test has been reported to be very low (0.2%) [87]. Cardiopulmonary tests, irrespective of the protocol, seemed to be well tolerated and safe in terms of severe complications. Exercise test is valuable for guiding the response to medication and invasive septal reduction therapies. Furthermore, CPET assessment and VO2max measurement are mandatory in the evaluation of end-stage candidates for heart transplant. 

ABPRE in patients between 18–40 years old and exercise NSVT have been associated with the occurrence of sudden death during follow up of patients with HCM. The use of ABPRE as a prognostic marker has been limited by lack of reproducibility of the variety of exercise protocols between clinical groups [87,99]. Current sudden death prediction scores do not include parameters of exercise performance [100]. Syncope but not dyspnea has been shown to identify patients at risk of an arrhythmic event. The pooled analysis presented here with up to 9145 patients from 23 publications, confirm the association between a reduced VO2max and cardiovascular outcomes and major arrhythmic events (Figure 7 and Table 3).

### 4.2. Exercise Protocols

One of the main conclusions of this systematic review is the demonstration of a wide variety of exercise protocols. This aspect, thus, deserves a dedicated discussion. The heterogeneity found in CPET methodologies hinders further comparisons of the results between studies. Other authors have criticized this matter previously, highlighting that CPET methodology is frequently selected based on convenience criteria due to the availability of specific equipment in laboratories [101]. Furthermore, previous investigations have found differences in the VO2max achieved in treadmill and cycle-ergometer, pointing out that a sudden intensity augment in the latter could lead to a premature ending of the test due to peripheral fatigue [102], an avoidable matter that can also occur following Bruce’s protocol in a treadmill [101]. At this point, ramp-like protocols with small intensity augments repeated each few seconds emerge as a suitable and more individualized option. These have been already used in the assessment of HCM patients with cycle-ergometers in few studies revised here [22,23,62], and also ramp-like tests have been recently validated in running settings [103].

Management of pharmacological therapy and previous food intake are also important in the performance of the test due to its potential effect on its development and outcomes. Drug management was heterogeneous, with a higher number of studies in which treatment was not withdrawn. This decision could influence different echo-electrocardiographic variables of the test which also have an effect on functional capacity. Moreover, meals prior to a functional assessment should also be taken into account due to the effect of intake on the exacerbation of symptoms of HCM symptoms [104]. LVOT increases with exercise in postprandial conditions [105,106]. It has also been associated with alterations in the hemodynamic response, failure to increase cardiac output, and increased pulmonary artery pressure and pulmonary capillary pressure [107].

On the other hand, drug–food interactions may happen, as it occurs in antihypertensive patients, which decrease serum zinc levels and increase glucose content [108,109]. Only 1 of the 69 studies reviewed reported withdrawal from alcohol 24 h before, while 3 indicated withdrawal from coffee but did not report on other stimulating beverages such as tea.

### 4.3. Extracardiac Factors and Functional Capacity

The impact of the extracardiac factors in the functional limitation is often underestimated. The average BMI of the population studied in all the publications stood at a value considered as overweight [110]. The prevalence of diabetes was similar to that found in general population, while that of dyslipidemia was higher [111,112]. This fact is indicative of the need to attend the bodyweight of HCM patients and could be related to restrictions in physical activity. Few studies include any extracardiac variables related to nutritional status, and none of them had the word ‘nutrition’ in their full texts. Hypertension and smoking habit were screened in 21 and 8 investigations, respectively. The first has been related to an increased odds ratio for reduced functional capacity, while no independent effect was observed for smoking habit [77].

It has been shown that diabetes (OR: 2.84; 95% CI: 1.89–4.25) and the history of hypertension (OR: 1.71; 95% CI: 1.32–2.23) have a negative effect on functional capacity, consisting in independent predictors of a value lower than 4 METs [77], while a higher hemoglobin content proved to be a protective factor (OR: 0.76; CI 95%: 0.65–0.68). In another study on which patients’ VO2max was compared after stratifying by their BMI, a reduction in VO2max was observed as BMI increased, with no differences between groups in cardiac variables [48]. Authors suggest that the primary limitation to exercise in overweight HCM patients may not be cardiac in origin. Olivotto and his collaborators had previously shown that obesity is an independent risk factor associated with a phenotypic progression to severe stages with the occurrence of adverse cardiac remodeling and in-creased left ventricular mass [113]. Furthermore, they suggest that obesity could modulate the expression of HCM genotype disease with severe symptoms and promote the development and progression of symptoms of heart failure.

### 4.4. Exercise Prescription

Physical exercise has emerged in the last decade as a non-pharmacological, non-surgical and safe alternative for HCM treatment, being especially effective in improving functional capacity in patients below the threshold of 7 METs [114]. Thus, various training protocols at intensities between 50 and 80% of heart rate reserve have suggested benefit effect on the VO2max in individuals with HCM [115,116]. These results suggest the need to maintain physical exercise as part of the treatment. Furthermore, a reduction in body mass index has recently been associated with greater functional capacity [48].

Exercise prescription in patients with HCM has been derived from a wide range of variables and different protocols. Lack of uniformity in the interpretation and adaptation of different protocols can hamper the eligibility of the best test for an individual patient. Lack of clarity on exercise prescription in HCM can also promote sedentarism and an overzealous attitude amongst physicians who worry on the risk of sudden cardiac death.

### 4.5. Future Perspectives

Understanding of physical response to exercise in HCM and the response to therapies has regain interest as new promising pharmacological agents have irrupted in the scene. Mavacamten, a recently developed myosin inhibitor, has demonstrated to increase exercise parameters and improve self-reported dyspnea class in patients with obstructive and non-obstructive HCM [117,118].

A new paradigm shift based on recent evidence that physical training can safely improve functional capacity in HCM patients is included in a recently published expert consensus paper on recommendations for physical activity in Cardiomyopathies [119]. Engagement in regular physical activity improves not only quality of life in patients with cardiomyopathies but also can prevent cardiovascular complications such as ischemic heart disease and should be encouraged [120]. Not only functional capacity considered as cardiovascular aerobic performance in an incremental test, but also muscular strength might contribute to quality of life and health benefits [121]. Especially when considering both together, concurrent aerobic and resistance training have shown beneficial effects in cardiac patients with pathologies that share some phenotypical characteristics from HCM [122].

Every patient with HCM requires a systematic individualised assessment. Exercise and lifestyle advice need to be age appropriate and tailored to the patient. While prevention of sudden cardiac death due to HCM overwhelmingly leads the initial management of the patients, prevention of cardiovascular risk factors and loss of years of life due to sedentarism needs to be balanced at the time of each assessment. Physiological evaluation of patients with HCM can be complicated by the interpretation of the results on different protocols and under different circumstances. Better understanding on the adequacy of the physiological testing and its limitation will empower both patients and physicians to move from a strictly conservative approach to a more consensual and patient centred evaluation.

### 4.6. Limitations

There are inherent limitations of the study. Despite systematic evaluation of the publications some of the studies reporting relevant information on functional assessment might not be included in the analysis. Part of the cohorts might be shared in different studies. Information from publications did not allow a dedicated analysis of subgroups regarding gender, symptoms (presence of syncope, self-reported NYHA class), diastolic function and heart rhythm (sinus rhythm vs. atrial fibrillation). Heterogenicity of the CPET protocols and cohorts limit analysis and conclusions.

## 5. Conclusions

CPET is a valuable tool and can safely perform for assessment of physical functional capacity in patients with HCM. VO2max is the most common performance measurement evaluated in functional studies, showing higher values in those based on cycle-ergometer compared to treadmill. A variety of protocols and the lack of standard protocol difficult comparisons between studies. Subgroup analysis shows that exercise intolerance seems to be more related to age, medication and comorbidities than HCM phenotype itself. Lower VO2max is consistently seen in HCM patients at risk of major cardiovascular outcomes.

## Figures and Tables

**Table 3:** Univariate hazard ratio of functional assessment derived parameters for composite endpoints.

Author, Year	VO2max	AT VO2	METs	% VO2	ABPRE	Arrhythmia
Coats, 2014 [28]	0.79 (0.74–0.83)	0.71 (0.62–0.82)	-	-	2.15 (1.58–2.94)	1.66 (1.23–2.23)
Desai, 2014 [32]	-	-	0.73 (0.63–0.85)	-	1.96 (0.20–3.27)	1.25 (0.51–3.10)
Feneon, 2016 [86]	-	-	0.99 (0.96–1.02)	-	-	-
Finocchiaro, 2015 [37]	0.52 (0.45–0.60)	-	-	5.36 (1.96–14.6) ^†^	-	-
Ghiselli, 2019 [39]	-	-	0.68 (0.53–0.87)	-	-	-
Gimeno, 2009 [87]	-	-	-	-	-	2.18 (1.07–4.45)
Magri, 2016 [55]	0.91 (0.85–0.98)	0.82 (0.72–0.94)	-	0.96 (0.93–0.99)	2.33 (1.003–5.4)	3.13 (1.38–7.01)
Magri, 2016 [89]	0.80 (0.76–0.84)	0.81 (0.74–0.88)	-	0.94 (0.92–0.95)	4.49 (2.91–6.93)	-
Magri, 2018 [56]	0.85 (0.81–0.89)	-	-	0.95 (0.94–0.97)	3.50 (2.22–5.31)	3.00 (1.22–7.39)
Masri, 2015 [90]	-	-	-	0.96 (0.93–0.98)	-	-
Michaelides, 2009 [91]	-	-	-	-	3.6 (0.8–15.6)	-
Moneghetti, 2017 [63]	0.57 (0.42–0.76)	-	0.55 (0.41–0.75)	0.59 (0.46–0.76)	-	-
Olivotto, 1999 [93]	-	-	-	-	4.50 (1.1–20.1)	-
Peteiro, 2012 [67]	-	-	0.74 (0.63–0.86)	-	-	3.13 (1.128.71)
Sorajja, 2012 [97]	0.92 (0.89–0.96)	0.99 (0.99–0.99)	0.81 (0.71–0.93)	0.98 (0.96–0.99)	-	-

VO2max: peak oxygen consumption, ATVO2: oxygen consumption at the anaerobic threshold, METs: metabolic equivalents, %VO2: percentage of age-gender predicted VO2max, ABPRE: abnormal blood pressure response to exercise. ^†^: univariate hazard ratio for reaching composite endpoints when %VO2 is <80%.

## Data Availability

The data presented in this study are openly available on the studies referenced in the figures, and individual data of each can be consulted in the original manuscripts.
